# Irritability of the Bladder Treated by Dilatation of the Urethra

**Published:** 1876-05

**Authors:** 


Irritability of the Bladder Treated by Dilatation of the Urethra.—In the *London Lancet* for March, Dr. H. Bendelack Hewetson records a case of great interest to the profession, as demonstrating the entire success of a mode of treatment recently introduced. The patient was a female aged 36, who for fifteen years had been under the necessity of rising "every half hour or hour" during the night to urinate. The urine passed in small quantities with great effort and pain, and there was a constant sense of weight and bearing down. Retention of urine came on occasionally, requiring the catheter. Her general health was much broken. On examination, the orifice of the urethra was found surrounded by warty growths. Under the influence of chloroform these were removed, and also a small hemorrhoidal tumor, and at the same time the sphincter ani was forcibly dilated by the fingers. Partial relief followed, but the relief was temporary. Dr. Hewetson placed her again under chloroform, and introduced the dilator, and slowly stretched the urethra so as to admit of the introduction of the forefingers into the bladder; distending not only the canal but the neck of the bladder. No bad effects of any kind followed, and for the first time for years she was not disturbed during the whole night. Her painful and distressing condition was entirely removed within a week. She gained strength and health rapidly. No return of the disease occurred. In six weeks she took a walk of eight miles without suffering. In short the cure was complete and permanent. "Thus then," says the writer in conclusion, "were the miserable and intractable sufferings of years, shutting out this poor woman alike from society and employment, put an end to by an operation whose best recommendation is its simplicity and success."

